# The Queensland high risk foot form (QHRFF) – is it a reliable and valid clinical research tool for foot disease?

**DOI:** 10.1186/1757-1146-7-7

**Published:** 2014-01-28

**Authors:** Peter A Lazzarini, Vanessa Ng, Ewan M Kinnear, Maarten C Kamp, Suzanne S Kuys, Cameron Hurst, Lloyd F Reed

**Affiliations:** 1 Allied Health Research Collaborative, Metro North Hospital & Health Service, Queensland Health, Rode Road, Chermside, Brisbane, QLD 4032, Australia; 2 Department of Podiatry, Metro North Hospital & Health Service, Queensland Health, Brisbane, Australia; 3 School of Clinical Sciences, Queensland University of Technology, Brisbane, Australia; 4 School of Medicine, The University of Queensland, Brisbane, Australia; 5 Department of Endocrinology, Metro North Hospital & Health Service, Queensland Health, Brisbane, Australia; 6 Centre for Musculoskeletal Research, Griffith Health Institute, Griffith University, Gold Coast, Australia; 7 Clinical Epidemiology Unit, Faculty of Medicine, Khon Kaen University, Khon Kaen, Thailand; 8 Institute of Health and Biomedical Innovation, Queensland University of Technology, Brisbane, Australia

**Keywords:** Foot, Disease, Tool, Valid, Reliable

## Abstract

**Background:**

Foot disease complications, such as foot ulcers and infection, contribute to considerable morbidity and mortality. These complications are typically precipitated by “high-risk factors”, such as peripheral neuropathy and peripheral arterial disease. High-risk factors are more prevalent in specific “at risk” populations such as diabetes, kidney disease and cardiovascular disease. To the best of the authors’ knowledge a tool capturing multiple high-risk factors and foot disease complications in multiple at risk populations has yet to be tested. This study aimed to develop and test the validity and reliability of a Queensland High Risk Foot Form (QHRFF) tool.

**Methods:**

The study was conducted in two phases. Phase one developed a QHRFF using an existing diabetes foot disease tool, literature searches, stakeholder groups and expert panel. Phase two tested the QHRFF for validity and reliability. Four clinicians, representing different levels of expertise, were recruited to test validity and reliability. Three cohorts of patients were recruited; one tested criterion measure reliability (n = 32), another tested criterion validity and inter-rater reliability (n = 43), and another tested intra-rater reliability (n = 19). Validity was determined using sensitivity, specificity and positive predictive values (PPV). Reliability was determined using Kappa, weighted Kappa and intra-class correlation (ICC) statistics.

**Results:**

A QHRFF tool containing 46 items across seven domains was developed. Criterion measure reliability of at least moderate categories of agreement (Kappa > 0.4; ICC > 0.75) was seen in 91% (29 of 32) tested items. Criterion validity of at least moderate categories (PPV > 0.7) was seen in 83% (60 of 72) tested items. Inter- and intra-rater reliability of at least moderate categories (Kappa > 0.4; ICC > 0.75) was seen in 88% (84 of 96) and 87% (20 of 23) tested items respectively.

**Conclusions:**

The QHRFF had acceptable validity and reliability across the majority of items; particularly items identifying relevant co-morbidities, high-risk factors and foot disease complications. Recommendations have been made to improve or remove identified weaker items for future QHRFF versions. Overall, the QHRFF possesses suitable practicality, validity and reliability to assess and capture relevant foot disease items across multiple at risk populations.

## Background

Foot disease contributes to considerable hospitalisation [1-4], amputation [5-8], institutionalisation [9,10], and death [2,11]; yet foot disease complications and these outcomes are largely preventable [5,6,12,13]. “High risk factors” (such as peripheral neuropathy, peripheral arterial disease or foot deformity) significantly increase the risk of developing “foot disease complications” (such as foot ulcers, infection or ischaemia) [1,14-16]. Diabetes populations are frequently acknowledged as the leading “at risk population” for foot disease due to the increased risk of developing high risk factors, and in turn foot disease complications, from diabetes [5-8,17]. However, other chronic disease populations (such as chronic kidney disease [15,16], cardiovascular disease [5,6,18,19] and some cancers [5,6,8]) have now been shown to cause comparable rates of high risk factors and foot disease complications to diabetes, and thus, are also becoming more readily identified as “at risk populations” for foot disease.

Best practice foot disease management has been shown to significantly reduce hospitalisation, amputation, mortality and overall costs within different at risk populations [5,12,13,20]. These multi-faceted best practice interventions commonly include screening for high-risk factors, multi-disciplinary management of foot disease complications, clinical training, evidence-based clinical pathway utilisation and regular capture and analysis of foot disease clinical data [5,6,12,13,20]. In consideration of the growing problem of foot disease, and the potential future improvements observed with best practice clinical management and research, it is imperative that any clinical tools to assess, capture, measure or analyse patient outcomes in at risk populations are valid and reliable.

There are a multitude of studies that have investigated a specific high risk foot factor or foot disease complication within multiple at risk populations [3,5,6,21]. Furthermore, numerous studies have investigated multiple high risk factors and foot disease complications in specific at risk populations such as diabetes [2,15,17]. However, very few studies have investigated multiple high risk foot factors and foot disease complications in multiple at risk populations. This situation appears to be mirrored by the clinical tools available to measure and report on foot disease. For example many validated single-item tools are available which measure specific high-risk factors or foot disease complications within multiple at risk populations [22-24]; such as the ankle brachial index to capture and measure peripheral arterial disease within various at risk populations [22]. Furthermore, many validated multi-item tools measuring multiple high-risk factors and foot disease complications in specific at risk populations have been developed [15,25-28]; such as the University of Texas Diabetic Foot Classification System to capture and measure multiple high risk factors and foot disease complications in people with diabetes only [25]. However, to the best of the authors’ knowledge a multi-item tool designed to measure multiple high-risk factors and foot disease complications in multiple at risk populations is yet to be developed and tested for validity and reliability.

Recently a Diabetic Foot Form (DFF) tool was developed to measure multiple high-risk factors and foot disease complications in people with diabetes only in diverse Queensland (Australia) settings [29]. The DFF was a multi-item tool developed from a number of existing clinical tools recommended in the literature to reliably capture various high-risk factors and foot disease complications in the diabetes population [29]. The implementation of the DFF in diverse Queensland clinical sites, in conjunction with other multi-faceted strategies, resulted in improved capture, measurement and management of high risk factors and foot disease complications and a corresponding reduction in outcomes such as hospitalisation and amputation [29,30]. However, the tool was not tested for validity or reliability and was designed only to measure multiple high-risk factors and foot disease complications in the specific at risk population of people with diabetes.

In this study, we sought to modify the DFF tool to enable the measurement of multiple high risk factors and foot disease complications in multiple at risk populations. It was intended that the new multi-item tool would align with best practice principles for clinical tools including being easily interpreted, practical to use and possessing high face, content and criterion validity, and inter- and intra-rater reliability [31-33]. Thus, the aims of this study were firstly to develop a multi-item Queensland High Risk Foot Form (QHRFF) tool to capture multiple high risk factors and foot disease complications in multiple applicable at risk populations, and secondly, to investigate the validity and reliability of the tool’s individual items when used by clinicians representing different levels of foot disease expertise.

## Methods

The study was conducted in two phases. Phase one involved development of the Queensland High Risk Foot Form (QHRFF) tool and phase two tested the validity and reliability of the QHRFF tool. Approval was granted from Institutional Ethics Committees and informed consent was obtained from all individual participants (patients and clinicians) for this study.

### Phase one – development of the tool

Phase one primarily aimed to select items for the development of a QHRFF tool that were practical to collect in an Australian clinical setting, applicable to multiple 'at risk’ populations, and provided high face and content validity. The overarching procedures used for item selection included using the original DFF [29] as the starting tool to build the new QHRFF, searching the electronic literature for other recommended foot disease tools, establishing an expert panel to guide development and using several rounds of stakeholder consultation to refine the tool.

The original DFF contained 64 items pertaining to the construct of foot disease in diabetes populations [29] and was used as the starting tool to modify into the QHRFF. The DFF was originally developed using similar procedures to those used in this study and appeared to possess high practicality, face and content validity [29]. Furthermore, the DFF had been routinely used in over 25 High Risk Foot Service sites throughout Queensland for three years to collect standard clinical data on diabetes-related foot disease [30].

An electronic literature search was undertaken of relevant electronic databases including MEDLINE (all years to June 2011), CINAHL (all years to June 2011) and relevant professional websites to identify existing foot disease-related tools. The basic terms searched included tools to identify peripheral neuropathy, peripheral arterial disease, foot deformity, foot ulceration, foot infection and amputation or synonyms.

An expert panel was established from members of the Queensland Statewide Diabetes Foot Working Group; a working group of the Queensland Statewide Diabetes Clinical Network. The panel comprised 14 expert clinicians, with between five and 25 years of experience in the area of foot disease management, from the fields of endocrinology, vascular surgery, podiatry, nursing, public health, quality improvement and research. The panel had the responsibility to decide upon the final items for the QHRFF tool after each round of consultation.

Refinements of the tool were achieved through numerous rounds of consultation and trialling of draft QHRFF versions with external stakeholders in relation to practicality, face and content validity. Stakeholders included up to 200 multidisciplinary professionals from sites registered to use the existing DFF, members of the Queensland Health Statewide Diabetes Clinical Network, Statewide Renal Clinical Network and Statewide Podiatry Network.

At the conclusion of phase one the expert panel and aforementioned network’s management committees determined, via consensus endorsement, that each item of the QHRFF possessed high practicality, face and content validity, and was applicable across multiple 'at risk’ populations (particularly diabetes, cardiovascular disease and chronic kidney disease) in Australian clinical settings. See Phase One Results for further details regarding the final QHRFF.

### Phase two – validity and reliability

The final endorsed QHRFF tool developed in phase one was then tested for validity and reliability. The general procedure for testing involved using podiatrists with different levels of foot disease expertise to assess patients with different levels and severity of high risk factors and foot disease. The authors decided to test only QHRFF items that directly related to the foot disease construct, thus, excluding items such as patients’ name, and facility. To test the validity of each item a general criterion measure (the agreement between two 'experts’) was initially assessed for reliability. Each item was then tested, using the podiatrists with different experience levels, against the criterion measure to evaluate magnitudes of concurrent criterion validity. The inter- and intra-rater reliability was also tested for the level of agreement on each item by podiatrists with different levels of experience.

#### Setting and participants

The testing was conducted within the High Risk Foot Services (HRFS), Metro North Hospital and Health Service, Brisbane, Australia. Eligible clinician participants were recruited from podiatrists practicing a minimum of one session per week in a HRFS. Four podiatrists were chosen as representative of the majority of clinicians managing foot disease within Queensland Health, and thus, potentially using the QHRFF in future. For the purposes of the study, levels of expertise were categorised using the Queensland Health 'Health Practitioner award’ [34]. Thus, expert clinicians were defined as either a 'consultant clinician’ (level 6) or 'specialist clinician’ (level 5) [34] and working in an acute hospital setting, plus, a member of the expert panel to ensure they understood the original intended construct for the QHRFF tool. General clinicians were defined as a 'senior clinician’ (level 4) or 'clinician’ (level 3) [34], working in a community setting, and thought to be representative of the general podiatry clinical workforce managing foot disease in Queensland. One of each level was recruited; one level 6, one level 5, one level 4 and one level 3 (however, the level 3 had been acting in a level 4 position at the time of the study). Written informed consent was obtained from all participants (patients and clinicians) prior to commencement of their study participation.

Eligible patient participants were consecutively recruited from patients already attending Community HRFS clinics for the care of high risk factors or foot disease complications; defined as a previous or current foot ulcer. Exclusion criteria included patients with a cognitive deficit, signs or symptoms of a systemic infection, younger than 18 years of age, or unwilling to provide written consent to participate. The authors considered that patients with previous or current foot ulcers would ensure that the majority of high risk factors and foot disease items had the realistic possibility of being present or absent, and thus, could be suitably tested on each participant. Furthermore, it was thought this population should possess the moderate prevalence rates, yet unpredictable mixes and severities of each item, of different high risk factors and foot disease complications, that are suggested in the literature to improve statistical robustness for validity and reliability studies [31].

Three different patient cohorts were used; one cohort to test the reliability of the criterion measures (agreement between 'experts’) (n = 32), another cohort to simultaneously test the criterion validity (an 'expert’ diagnosis against general clinicians) and inter-rater reliability (n = 43), and the last cohort to test the intra-rater reliability of a general clinician (n = 19). The recruitment of consecutive community patients did mean that patients may have been familiar to the level 3 or level 4 clinicians; however, not to the expert 'gold standard’ clinicians working in the hospital setting. To minimise the risk of patients being familiar to the level 3 or level 4 clinicians’ seven different Community HRFS clinics were used to recruit patient participants. The level 3 and level 4 clinicians had only worked at two of the seven clinics recruiting patients.

#### Procedures

A designated research assistant coordinated all procedures. Training of each clinician consisted of being provided with a QHRFF manual that gave a definition of each item [35], a 1–2 hour training session on instructions and tips to use the QHRFF tool, and each was encouraged to trial the tool on their existing patients and clarify any queries with the research assistant prior to testing.

The general assessment procedure for each validity or reliability test consisted of patients having their feet examined by at least two different clinicians, blinded from each other’s assessment, within the one clinical visit (validity and inter-rater reliability). However, to ensure all clinicians had an equal opportunity to determine the patient’s debridement and wound management needs, all were permitted to visually inspect the patient’s feet together for up to five minutes prior to any clinician ratings. In this initial inspection period, the clinicians were instructed to only visually inspect the need for debridement and previous wound dressings whilst not conversing with or touching the patient or each other. The order of clinician assessment after this inspection period was then at the discretion of the research assistant based on clinician availability. The first clinician would have the additional task of debriding the wound or callus if they deemed necessary and the last clinician the additional task of completing any clinical management.

The research assistant ensured all clinicians were blinded from each other’s assessments in separate rooms and that all examination records were de-identified. Each clinician conducted the assessment of patient’s feet using the QHRFF to record their assessment and management recommendations. Demographic, medical history and medication information were available from the patients’ medical records or by direct communication with the patients themselves. To minimise assessment bias all historical foot-related records (including progress notes, DFF tools or QHRFFs tools) were removed from the medical record prior to each clinician’s assessment.

##### Criterion measure

The criterion measure was tested on the first cohort of patients. The criterion measure (i.e. 'gold standard’ diagnosis) for each QHRFF item in this study was defined by the authors as the diagnosis made by an expert clinician. Expert clinicians were pragmatically chosen as a general criterion measure for all items, instead of using multiple resource and time intensive individual gold standard-recognised criterion measures (such as nerve conduction studies for neuropathy), due to the resource and time constraints of the study. Best practice dictates that any criterion measure should be reliable, free from bias and measure the same item as the new tool [31]. Thus, the study’s criterion measure, of an expert clinician, required testing to determine its reliability, independence (free from bias) and applicability, prior to its use as a 'gold standard’ criterion measure in the criterion-related validity tests. The authors tested the criterion measure by testing the reliability of the agreement between two independent (blinded from one another) experts (one level 6 and one level 5) to determine the magnitude of reliability of agreement on their 'gold standard’ diagnoses for each item on the same patient cohort. The “general assessment procedure” as described above was utilised for each patient. Once the criterion measure for each item was determined to be reliable, the criterion validity of each QHRFF item was tested.

##### Criterion validity

The criterion validity and inter-rated reliability was then tested simultaneously on a second cohort of patients. Criterion validity was tested using the concurrent criterion validity method by comparing the reliable criterion measure (one of the expert clinicians) against each of the two representative 'general’ clinicians (one level 3 and one level 4). Thus, two separate criterion assessments were carried out for each item; one using the level 3 clinician against the criterion measure, and another the level 4 clinician against the criterion measure. Again the “general assessment procedure” as described above was utilised for each patient when testing for criterion validity.

##### Inter-rater reliability

Inter-rater reliability tests were performed simultaneously with the criterion validity tests on the same second cohort of patients and were tested using the expert clinician (level 5), senior clinician (level 4) and clinician (level 3). Thus, three inter-rater reliability measures of agreements were carried out for each item; one testing the agreement between the level 5 and level 4 clinician, a second between the level 5 and level 3 clinician, and a third between the level 4 and level 3 clinician. “General assessment procedures” as described above were again utilised for each patient when testing for inter-rater reliability.

##### Intra-rater reliability

Intra-rater reliability was tested on a third cohort of patients. The clinician with the least expertise (level 3) was used to test intra-rater reliability as it was hypothesised that the clinician with the least expertise would have the most variability of the tested clinicians. The two different time points to test intra-rater reliability were between one and four weeks apart. This time period was chosen as it was considered that a minimum of one week (of full clinical load) would be necessary to adequately reduce recall bias in a clinician, plus, a maximum of four weeks would not be sufficient time for the majority of items to markedly change and thus items would remain stable between ratings. Any items that did not fit this criteria were excluded from intra-rater testing. Furthermore, this time period aligned with any necessary follow up treatment time period for patients, and thus, was convenient to patient participants. To control for any potential changes in foot ulcer characteristics over time (for example ulcer combined surface area and clinical signs of infection) digital images were used. Digital photographic images taken of foot ulcers at the first rating were used at the second rating to standardise the foot ulcer characteristics across two time points [36]. Images were taken perpendicular and 30-60 cm away from ulcers at the first rating, and incorporated two calibrated measures across the length and width of the ulcer in the image. These images were reviewed by the clinician at the second rating to determine the foot ulcer characteristics only.

### Statistical analysis

All data was analysed using SPSS 19.0 for Windows (SPSS Inc., Chicago, IL, USA) or GraphPad Software. Descriptive statistics were used to display the single demographic, medical co-morbidity, high-risk factors and foot disease variables for each cohort of patient participants; using means and standard deviations (SD) for continuous variables (which were normally distributed) or proportions for categorical variables. Each above descriptive cohort variable was collected from the clinician with most expertise or on the first rating of the intra-rater reliability cohort. Chi-squared test of independence and ANOVA were used to test for differences between the three cohorts’ characteristics. A significance level of p < 0.05 was used throughout.

All QHRFF items were tested for validity and reliability; except those stated items considered not to impact on the foot disease construct. Criterion validity was tested by calculating sensitivity, specificity and positive predictive values. Ordinal variables were collapsed into dichotomous data to enable calculations of sensitivity, specificity and positive predictive values. All measures of agreements between clinicians were tested using Kappa (K) for dichotomous variables, weighted Kappa (wK) for ordinal variables and intra-class correlation (ICCs) (model 2, 1) for continuous variables [31,37,38]. Kappa and wK value (Standard Errors (SE)) strengths were categorised as: no agreement < 0; slight agreement = 0 – 0.20; fair agreement = 0.21 - 0.40; moderate agreement = 0.41 – 0.6; substantial agreement = 0.61 – 0.8; and near perfect agreement = 0.81 – 1.0 [31,37,38]. ICC (SD) strengths were categorised as: weak-moderate agreement < 0.75 and strong agreement > 0.75 [31].

## Results

### Phase one – development of the tool

The literature search identified 174 papers that reported on tools to identify or measure foot disease complications and/or high-risk factors, with the majority of papers specifically relating to diabetes populations (90 (52%)). Tools reported in other populations were in conditions commonly identified in the literature to be associated with lower limb amputation [5-8]. These included cardiovascular disease (peripheral arterial disease), chronic kidney disease, malignancy, infection and other neurological conditions [5-8]. No tool was identified that was specifically designed to identify multiple high-risk factors and foot disease complications in multiple at risk populations.

Fifty-nine individual tools were identified from the literature search; 23 were considered to have adequate practicality, face validity and applicability to an Australian clinical context to be considered for the QHRFF. These tools and the original DFF tool [29] were considered by the expert panel for QHRFF item selection. At this point, the expert panel determined that the tool should be divided into a clinical assessment record section that informed a separate data collection section, and thus, only the data collection section would require testing.

**Figure 1:**
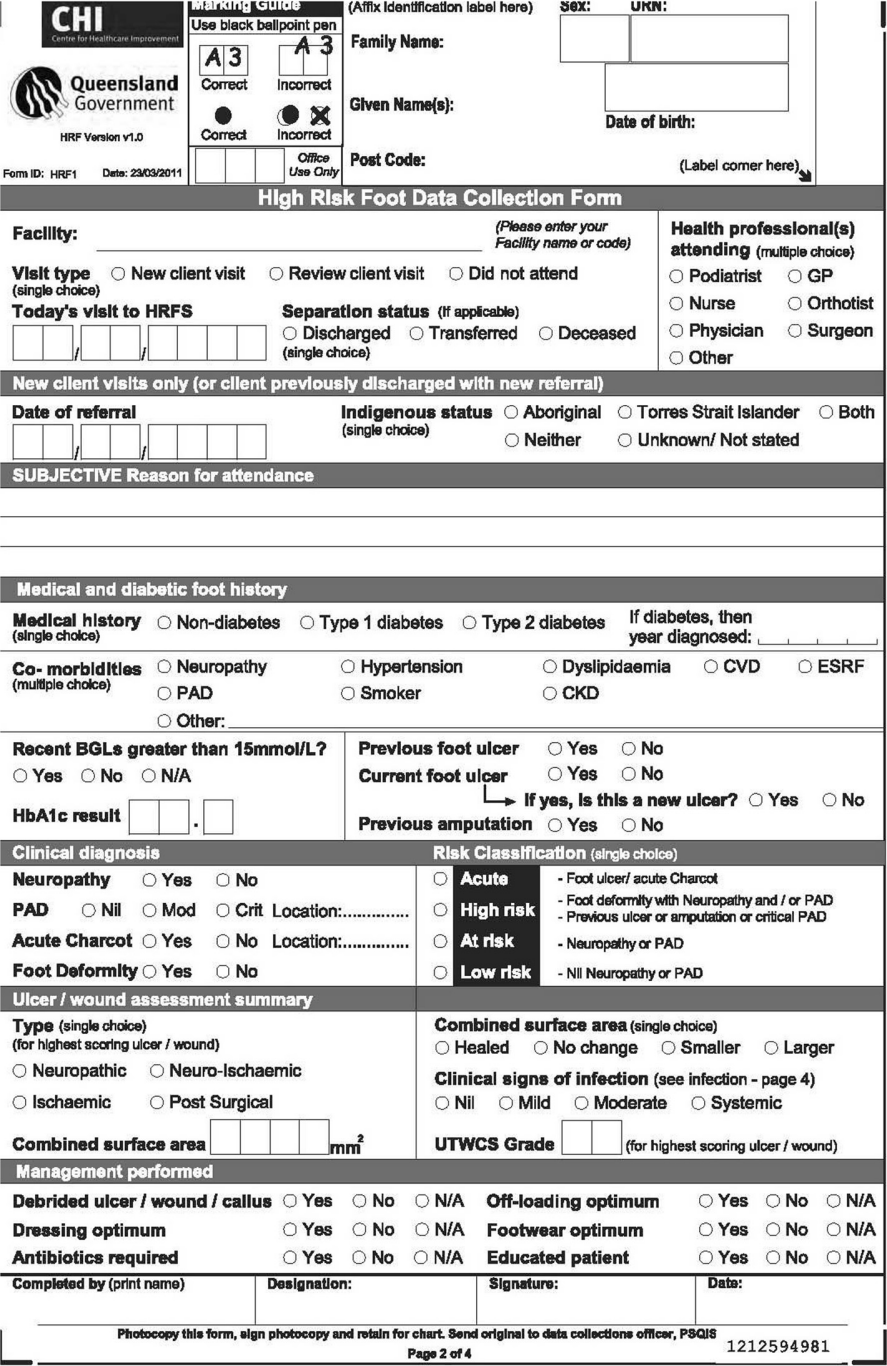
Queensland high risk foot data collection form (QHRFF).

Overall 87 items were initially identified from the considered tools. At the conclusion of phase one procedures the final endorsed QHRFF data collection tool was made up of 46-items (excluding general identification items) covering seven domains (Figure 1). The seven content domains included identifying general demographics, different health professionals attending, medical co-morbidity history, high-risk factor history, clinical diagnosis of high-risk factors, clinical diagnoses of foot disease complications, and clinical management principles performed. A QHRFF manual was developed to provide definitions and instructions on each item contained in the tool [35]. Table 1 outlines the tools or literature used to support each QHRFF item. At the conclusion of phase one, the expert panel concluded that the QHRFF tool’s items had the required high clinical practicality, applicability to multiple at risk populations, and good face and content validity. Subsequently, the tool was endorsed for use by the Queensland Health Statewide Diabetes, Renal and Podiatry Clinical Networks. Thus, the tool was ready for validity and reliability testing.

**Table 1:** Supporting tools or literature for QHRFF items

**QHRFF item**	**Supporting tool or literature**
General demographics*	[35]
Indigenous status	[14]
Health professionals attending*	[14,39]
Medical co-morbidity history*	[40,41]
Medical (diabetes) history	[5-8]
Diabetes year diagnosed (duration)	[17,42]
Recent BGLs > 15 mmol/L	[41,42]
HbA1c result	[41,42]
CKD	[41,43]
ESRF	[41,43]
High-risk factor history*	[14,35,39]
**Clinical diagnoses of high-risk factors**	
Neuropathy	Monofilament test [1,14,23,39,44,45]
PAD	Foot pulses, ankle brachial index and/or toe systolic pressure [1,14,21,22,39,46-49]
Acute Charcot	[14,39]
Foot deformity	Six-point foot deformity scale [14,50]
Risk classification	[14,39,51,52]
**Clinical diagnoses of foot disease**	
(Ulcer) type	[14,53,54]
Combined surface area mm^2^	[54-56]
Combined surface area (change since last visit)	[55-59]
Clinical signs of infection	[1,54,60,61]
UTWCS grade	UTWCS tool [14,62,63]
Ulcer depth#	[1,14,54]
Clinical management principles performed*	[14,39]

*All items in this Domain cite the same references, unless otherwise stated.

#Ulcer depth was not specifically an item recorded on the QHRFF, but can be directly extrapolated from the UTWCS Grade item.

### Phase two – validity and reliability

Forty items were tested for validity and reliability unless otherwise stated. The items not tested were those considered not to impact on the foot disease construct; i.e. 'facility’, 'visit type’, 'todays visit to HRFS’, 'separation status’, and 'date of referral’. Table 2 displays the general demographic, medical co-morbidity history, high-risk factor history, clinical diagnoses of high-risk factors and foot disease complications variable prevalence for the three patient cohorts used. No significant differences were noted for these variables, except for diabetes duration and any other co-morbidity (p < 0.05). All cohorts contained variables with moderate prevalence rates (> 15%) [31], except for different health professionals attending previously, co-morbidity of end stage renal failure (ESRF), acute Charcot and ischaemic ulcers.

**Table 2:** Demographic, co-morbidity and high-risk foot complication data for each patient cohort (n (%) unless otherwise stated)

	**Criterion measure**	Criterion validity* & inter-rater reliability*	**Intra-rater reliability**	**p Value**
**General demographics**				
Numbers	32	43	19	
Male	25 (78%)	37 (86%)	17 (90%)	0.501
Age, mean (SD) (years)#	69 (13)	68 (13)	70 (15)	0.826
Age range (years)	48 – 90	36 – 89	42 – 90	
Indigenous	1 (3%)	0 (0%)	1 (5%)	NA
**Health professionals attending (in the past week)**				
Podiatrist	32 (100%)	43 (100%)	19 (100%)	1.000
GP	2 (6%)	10 (23%)	2 (11%)	0.103
Nurse	3 (9%)	12 (28%)	4 (21%)	0.141
Orthotist	0	0	0	NA
Physician	2 (6%)	1 (2%)	1 (5%)	NA
Surgeon	1 (3%)	3 (7%)	0	NA
Other	0	0	0	NA
**Medical co-morbidity history**				
Diabetes (type 1 or 2)	28 (88%)	35 (81%)	16 (84%)	0.384
Type 2 diabetes	28 (88%)	31 (72%)	14 (74%)	0.256
Diabetes duration (years)#	12 (7)	23 (11)	20 (14)	0.001**
Recent BGLs > 15 mmol/L	5 (16%)	4 (9%)	5 (26%)	0.220
HbA1c (SD)#	9.2 (3.5)	8.1 (1.6)	8.4 (2.7)	0.450
Hypertension	28 (88%)	30 (70%)	11 (58%)	0.053
Dyslipidaemia	23 (72%)	25 (58%)	9 (47%)	0.201
Smoker	5 (16%)	11 (26%)	3 (16%)	0.492
CVD	21 (66%)	21 (49%)	9 (47%)	0.281
CKD	5 (16%)	13 (30%)	5 (26%)	0.339
ESRF	0	1 (2%)	0	NA
Other (non-listed)	29 (91%)	36 (84%)	11 (58%)	0.013**
**High-risk history**				
Neuropathy	31 (97%)	37 (86%)	18 (95%)	0.214
PAD	14 (44%)	21 (49%)	8 (42%)	0.853
Previous foot ulcer	27 (84%)	37 (86%)	17 (90%)	0.878
Current foot ulcer	32 (100%)	42 (98%)	19 (100%)	0.549
New foot ulcer	21 (66%)	42 (98%)	19 (100%)	≤ 0.001**
Previous amputation	16 (50%)	17 (40%)	7 (37%)	0.566
**Clinical diagnoses of high-risk factors**				
Neuropathy	31 (97%)	38 (88%)	19 (100%)	0.146
Any PAD	14 (44%)	22 (51%)	8 (42%)	0.735
Acute Charcot	1 (3%)	1 (2%)	0	NA
Foot deformity	26 (81%)	33 (77%)	17 (90%)	0.501
Acute risk classification	32 (100%)	42 (98%)	19 (100%)	0.549
**Clinical diagnoses of foot disease**				
Ulcer type: neuropathic	14 (44%)	14 (33%)	8 (42%)	0.572
Ulcer type: neuroischaemic	9 (28%)	16 (37%)	8 (42%)	0.555
Ulcer type: ischaemic	0	3 (7%)	1 (5%)	NA
Ulcer type: other	9 (28%)	9 (21%)	2 (11%)	0.339
Combined surface area (mm^2^)#	122 (191)	352 (899)	379 (1436)	0.469
Any clinical infection	6 (19%)	14 (33%)	2 (11%)	0.125
UTWCS (Grade 1A)	13 (41%)	14 (33%)	7 (37%)	0.770
Deep ulcer (Grade 2 or 3)^	8 (25%)	6 (14%)	2 (11%)	0.394

* = Same participants used for three tests.

** = Statistically significant (p < 0.05).

# = Measure in mean (standard deviation).

^ = Deep ulcer determined from the UTWCS Grade item.

NA = Not applicable to test as the assumption of Chi-squared test is violated as 2 cells had expected counts of < 5.

GP = General Practitioner; CVD = Cardiovascular Disease; ESRF = End Stage Renal Failure; PAD = Peripheral Arterial Disease; UTWCS = University of Texas Wound Classification System.

#### Criterion measure

Table 3 displays the reliability results for the criterion measure of expert clinicians. Thirty-two items were able to be statistically tested. Nine items (28%) recorded near perfect categories of agreement, nine (28%) substantial/strong categories, eleven (34%) moderate categories and three (9%) weak/fair categories. Thus, overall 29 (91%) of these items recorded at least a moderate category of reliability (K > 0.4; ICC > 0.75). The items recording weaker categories of reliability included other (non-listed) condition, University of Texas Wound Classification System (UTWCS) grade and optimum footwear.

**Table 3:** Criterion measure reliability – measure of agreement between gold standard experts

**QHRFF item**	**%**	***K *** **(SE)**	**Strength of agreement [**[37]**]**
**General demographics**			
Indigenous status*	77.4	0.45 (0.17)	Moderate
**Health professionals attending (in the past week)**			
Podiatrist	96.9	--	--
GP	81.2	0.78 (0.21)	Substantial
Nurse	84.4	0.47 (0.18)	Moderate
Orthotist	100	--	--
Physician	96.9	0.78 (0.21)	Substantial
Surgeon	100	1.00 (0.01)	Near perfect
Other	100	--	--
**Medical co-morbidity history**			
Medical (diabetes) history	100	1.00 (0.01)	Near perfect
Diabetes duration#		0.94 (0.88–0.98)	Strong
Recent BGLs > 15 mmol/L*	90.6	0.81 (0.11)	Near perfect
HbA1c result#		0.93 (0.79–0.98)	Strong
Hypertension	87.5	0.60 (0.17)	Moderate
Dyslipidaemia	75.0	0.46 (0.16)	Moderate
Smoker	96.9	0.89 (0.11)	Near perfect
CVD	93.8	0.86 (0.10)	Near perfect
CKD	100	1.00 (0.01)	Near perfect
ESRF	96.9	--	--
Other (non-listed)	46.9	0.04 (0.09)	Slight
**High-risk history**			
Neuropathy	93.8	0.48 (0.31)	Moderate
PAD	84.4	0.68 (0.13)	Substantial
Previous foot ulcer	84.4	0.45 (0.21)	Moderate
Current foot ulcer	96.9	--	--
New foot ulcer	83.9	0.58 (0.15)	Moderate
Previous amputation	90.6	0.81 (0.10)	Near perfect
**Clinical diagnoses of high-risk factors**			
Neuropathy	96.9	0.65 (0.32)	Substantial
PAD*	77.4	0.61 (0.12)	Substantial
Acute Charcot	100	1.00 (0.01)	Near perfect
Foot deformity	81.3	0.46 (0.19)	Moderate
Risk classification*	96.8	--	--
**Clinical diagnoses of foot disease**			
Ulcer type*	87.5	0.85 (0.08)	Near perfect
Combined surface area mm^2^#		0.73 (0.52-0.86)	Moderate
Clinical signs of infection*	84.3	0.68 (0.15)	Substantial
UTWCS grade#		0.56 (0.28–0.76)	Weak-moderate
Ulcer depth^	78.1	0.67 (0.13)	Substantial
**Clinical management principles performed**			
Debrided ulcer/callus*	93.8	--	--
Dressing optimum*	96.9	--	--
Antibiotics required*	84.4	0.47 (0.19)	Moderate
Off-loading optimum*	68.8	0.41 (0.14)	Moderate
Footwear optimum*	65.6	0.28 (0.16)	Fair
Educated patient*	100	--	--

K = Kappa; (SE) = Standard Error; % = Percentage agreement.

-- = Measure of agreement was unable to be calculated because at least one variable was a constant.

GP = General Practitioner; CVD = Cardiovascular Disease; ESRF = End Stage Renal Failure; PAD = Peripheral Arterial Disease; UTWCS = University of Texas Wound Classification System.

* = Weighted Kappa used for ordinal data.

# = Intraclass correlation (ICC) (Standard Deviation (SD)) for continuous data.

^ = Depth was determined from the UTWCS score.

#### Criterion validity

Tables 4 and 5 display the criterion validity results for both the senior (level 4) clinician and clinician (level 3) respectively tested against the criterion measure for each item. Thirty-six items were able to be statistically tested for sensitivity, specificity or positive predictive values (PPV) on both clinicians. Thus, 72 different tests were each performed for sensitivity, specificity and PPV. Sixty-one (85%), 59 (82%) and 60 (83%) items recorded at least moderate categories (> 0.7) for sensitivity, specificity and PPV respectively. Conversely, three (8%), five (14%) and four (11%) out of the 36 items recorded weak categories (< 0.7) for sensitivity, specificity and PPV respectively on both clinicians tested. The items registering weak categories of validity (sensitivity, specificity or PPV on both clinicians) included identifying a patient that had attended a GP, physician or surgeon; chronic kidney disease (CKD) or other (non-listed) condition; previous, current and new foot ulcer all had poor specificity in particular; and optimum footwear had poor PPVs.

**Table 4:** **Criterion validity summary statistics for senior clinician (Sensitivity**, **specificity and positive predictive value**) & **inter**-**rater reliability between expert and senior clinicians** (**Kappa and strength of agreement**)

**QHRFF field**	**%**	***K *** **(SE)**	**Strength of agreement [**[37]**]**	**Sensitivity (95% CI)**	**Specificity (95% CI)**	**PPV (95% CI)**
**General demographics**						
Any indigenous status	100	--	--	--	1.00 (0.90-1.00)	--
**Health professionals attending (in the past week)**						
Podiatrist	100	--	--	1.00 (0.90-1.00)	--	1.00 (0.90-1.00)
GP	86.0	0.64 (0.14)	Substantial	0.80 (0.44-0.96)	0.88 (0.71-0.96)	0.67 (0.35-0.88)
Nurse	93.0	0.83 (0.09)	Near perfect	0.92 (0.60-1.00)	0.94 (0.77-0.99)	0.85 (0.54-0.97)
Orthotist	100	--	--	--	1.00 (0.90-1.00)	--
Physician	93.0	--	--	0 (0 – 0.95)	0.95 (0.83-0.99)	0 (0 – 0.80)
Surgeon	95.3	0.64 (0.24)	Substantial	0.67 (0.13-0.98)	0.98 (0.85-1.00)	0.67 (0.13-0.98)
Other	100	--	--	--	1.00 (0.90-1.00)	--
**Medical co**-**morbidity history**						
Any medical (diabetes) history	100	1.00 (0.01)	Near perfect	1.00 (0.88-1.00)	1.00 (0.60-1.00)	1.00 (0.88-1.00)
Diabetes duration#		0.90 (0.80– 0.95)	Strong			
Recent BGLs > 15 mmol/L	93.0	0.69 (0.16)	Substantial	0.57 (0.20-0.88)	1.00 (0.88-1.00)	1.00 (0.40-1.00)
HbA1c result#		1.00 (0.99–1.00)	Strong			
Hypertension	93.0	0.83 (0.09)	Near perfect	0.97 (0.81-1.00)	0.85 (0.54-0.97)	0.94 (0.77-0.99)
Dyslipidaemia	95.3	0.90 (0.07)	Near perfect	0.96 (0.78-1.00)	0.94 (0.71-1.00)	0.96 (0.78-1.00)
Smoker	90.7	0.72 (0.13)	Substantial	0.64 (0.32-0.88)	1.00 (0.87-1.00)	1.00 (0.56-1.00)
CVD	90.7	0.81 (0.09)	Near perfect	0.86 (0.63-0.96)	0.95 (0.75-1.00)	0.95 (0.72-1.00)
CKD	81.4	0.49 (0.15)	Moderate	0.46 (0.20-0.74)	0.97 (0.81-1.00)	0.86 (0.42-0.99)
ESRF	1.00	1.00 (0.01)	Near perfect	1.00 (0.05-1.00)	1.00 (0.90-1.00)	1.00 (0.05-1.00)
Other (non-listed)	57.1	0.09 (0.12)	Slight	0.57 (0.40-0.73)	0.57 (0.20-0.88)	0.87 (0.65-0.97)
**High**-**risk history**						
Neuropathy	93.0	0.73 (0.15)	Substantial	0.95 (0.80-0.99)	0.83 (0.36-0.99)	0.97 (0.84-1.00)
PAD	88.4	0.77 (0.10)	Substantial	0.86 (0.63-0.96)	0.91 (0.69-0.98)	0.90 (0.67-0.98)
Previous foot ulcer	79.1	0.28 (0.18)	Fair	0.84 (0.67-0.93)	0.50 (0.14-0.86)	0.91 (0.75-0.98)
Current foot ulcer	95.3	--	--	0.98 (0.86-1.00)	0 (0 – 0.95)	0.98 (0.86-1.00)
New foot ulcer	93.0	--	--	0.95 (0.82-0.99)	0 (0 – 0.95)	0.98 (0.86-1.00)
Previous amputation	97.7	0.95 (0.05)	Near perfect	0.94 (0.69-1.00)	1.00 (0.84-1.00)	1.00 (0.76-1.00)
**Clinical diagnoses of high**-**risk factors**						
Neuropathy	97.7	0.90 (0.10)	Near perfect	0.97 (0.85-1.00)	1.00 (0.46-1.00)	1.00 (0.88-1.00)
Any PAD	93.0	0.86 (0.8)	Near perfect	0.91 (0.69-0.98)	0.95 (0.74-1.00)	0.95 (0.74-1.00)
Acute Charcot	100	1.00 (0.01)	Near perfect	1.00 (0.05-1.00)	1.00 (0.90-1.00)	1.00 (0.05-1.00)
Foot deformity	86.0	0.51 (0.16)	Moderate	1.00 (0.87-1.00)	0.4 (0.14-0.73)	0.85 (0.69-0.94)
Acute risk classification	97.7	--	--	1.00 (0.90-1.00)	0 (0 – 0.95)	0.98 (0.86-1.00)
**Clinical diagnoses of foot disease**						
Ulcer type: neuropathic (neuropathic or neuroisch.)	90.7	0.76 (0.11)	Substantial	0.88 (0.72-0.96)	1.0 (0.63-1.00)	1.00 (0.86-1.00)
Ulcer type: ischaemic	79.1	0.58 (0.13)	Moderate	0.75 (0.51-0.90)	0.83 (0.60-0.94)	0.79 (0.54-0.93)
(Neuroisch. or ischaemic)						
Combined surface area mm^2^#		0.93 (0.87–0.96)	Strong			
Any clinical infection	88.4	0.72 (0.12)	Substantial	0.71 (0.42-0.90)	0.97 (0.80-1.00)	0.91 (0.57-1.00)
UTWCS (Grade 1A)	83.7	0.65 (12)	Substantial	0.85 (0.56-0.97)	0.83 (0.64-0.93)	0.71 (0.44-0.89)
Deep ulcer	95.3	0.81 (0.13)	Near perfect	0.71 (0.30-0.95)	0.97 (0.84-1.00)	0.83 (0.36-0.99)
(Grade 2 or 3)^						
**Clinical management principles performed**						
Ulcer debridement is required	95.3	0.66 (0.01)	Substantial	1.00 (0.89-1.00)	1.00 (0.20-1.00)	1.00 (0.89-1.00)
Dressing is optimum	100	--	--	1.00 (0.90-1.00)	--	1.00 (0.90-1.00)
Antibiotics are required	93.0	0.82 (0.10)	Near perfect	0.83 (0.51-0.97)	0.94 (0.77-0.99)	0.83 (0.51-0.97)
Off-loading is optimum	93.0	0.80 (0.11)	Substantial	0.80 (0.44-0.96)	0.97 (0.82-1.00)	0.89 (0.51-0.99)
Footwear is optimum	72.1	0.31 (0.16)	Fair	0.50 (0.20-0.80)	0.82 (0.64-0.92)	0.45 (0.18-0.75)
Educated patient	97.7	--	--	0.98 (0.86-1.00)	--	1.00 (0.90-1.00)

K = Kappa; (SE) = Standard Error; % = Percentage agreement; PPV = Positive Predictive Value.

-- = Measure of agreement was unable to be calculated because at least one variable was a constant.

GP = General Practitioner; CVD = Cardiovascular Disease; ESRF = End Stage Renal Failure; PAD = Peripheral Arterial Disease; UTWCS = University of Texas Wound Classification System.

# = Intraclass correlation (ICC) (Standard Deviation (SD)) for continuous data.

^ = Depth was determined from the UTWCS score.

**Table 5:** **Criterion validity summary statistics for clinician** (**Sensitivity**, **specificity and positive predictive value**) & **inter**-**rater reliability between expert and clinician** (**Kappa and strength of agreement**)

**QHRFF field**	**%**	***K *** **(SE)**	**Strength of agreement [**[37]**]**	**Sensitivity (95% CI)**	**Specificity (95% CI)**	**PPV (95% CI)**
**General demographics**						
Any indigenous status	100	--	--	--	1.00 (0.90-1.00)	--
**Health professionals attending (in the past week)**						
Podiatrist	88.4	--	--	0.88 (0.74-0.96)	--	0.88 (0.74-0.96)
GP	81.4	0.39 (0.17)	Fair	0.40 (0.14-0.73)	0.94 (0.78-0.99)	0.67 (0.24-0.94)
Nurse	79.1	0.37 (0.16)	Fair	0.33 (0.11-0.65)	0.97 (0.81-1.00)	0.80 (0.30-0.99)
Orthotist	100	--	--	--	1.00 (0.90–1.00)	--
Physician	97.6	0.66 (0.32)	Substantial	1.00 (0.05-1.00)	0.98 (0.86-1.00)	0.50 (0.03-0.97)
Surgeon	90.7	0.45 (0.23)	Moderate	0.67 (0.13-0.98)	0.93 (0.79-0.98)	0.40 (0.07-0.83)
Other	100	--	--	--	0.98 (0.86-1.00)	--
**Medical co**-**morbidity history**						
Any medical (diabetes) history	100	100 (0.01)	Near perfect	1.00 (0.88-1.00)	1.00 (0.60-1.00)	1.00 (0.88-1.00)
Diabetes duration#		0.95 (0.90–0.97)	Strong			
Recent BGLs > 15 mmol/L	90.7	0.62 (0.17)	Substantial	1.00 (0.40-1.00)	0.90 (0.75-0.97)	0.50 (0.17-0.83)
HbA1c result#		1.00 (0.99–1.00)	Strong			
Hypertension	86.0	0.68 (0.12)	Substantial	0.87 (0.68-0.96)	0.85 (0.54-0.97)	0.93 (0.75-0.99)
Dyslipidaemia	79.1	0.58 (0.12)	Moderate	0.76 (0.54-0.90)	0.83 (0.58-0.96)	0.86 (0.64-0.96)
Smoker	88.4	0.69 (0.13)	Substantial	0.73 (0.39-0.93)	0.94 (0.78-0.99)	0.80 (0.44-0.96)
CVD	79.1	0.58 (0.12)	Moderate	0.71 (0.48-0.88)	0.86 (0.64-0.96)	0.83 (0.58-0.96)
CKD	86.0	0.65 (0.13)	Substantial	0.69 (0.39-0.90)	0.93 (0.76-0.99)	0.82 (0.48-0.97)
ESRF	97.7	0.66 (0.32)	Substantial	1.00 (0.05-1.00)	0.98 (0.86-1.00)	0.50 (0.03-0.97)
Other (non-listed)	79.1	0.19 (0.19)	Slight	0.89 (0.73-0.96)	0.29 (0.05-0.7)	0.86 (0.70-0.95)
**High**-**risk history**						
Neuropathy	93.0	0.73 (0.15)	Substantial	0.95 (0.80-0.99)	0.83 (0.36-0.99)	0.97 (0.84-1.00)
PAD	86.0	0.72 (0.10)	Substantial	0.76 (0.52-0.91)	0.95 (0.75-1.00)	0.94 (0.69-1.00)
Previous foot ulcer	79.1	0.28 (0.18)	Fair	0.84 (0.67-0.93)	0.50 (0.14-0.86)	0.91 (0.75-0.98)
Current foot ulcer	97.7	--	--	1.00 (0.90-1.00)	0 (0 – 0.95)	0.98 (0.86-1.00)
New foot ulcer	97.4	--	--	0.90 (0.76-0.97)	0 (0 – 0.95)	0.97 (0.85-0.99)
Previous amputation	95.3	0.90 (0.07)	Near perfect	0.88 (0.62-0.98)	1.00 (0.84-1.00)	1.00 (0.75-1.00)
**Clinical diagnoses of high-risk factors**						
Neuropathy	100	1.00 (0.01)	Near perfect	1.00 (0.89-1.00)	1.00 (0.46-1.00)	1.00 (0.89-1.00)
Any PAD	95.3	0.91 (0.06)	Near perfect	0.91 (0.69-0.98)	1.00 (0.81-1.00)	1.00 (0.80-1.00)
Acute Charcot	100	1.00 (0.01)	Near perfect	1.00 (0.05-1.00)	1.00 (0.90-1.00)	1.00 (0.05-1.00)
Foot deformity	93.0	0.80 (0.11)	Substantial	0.97 (0.82-1.00)	0.80 (0.44-0.96)	0.94 (0.79-0.99)
Acute risk classify	97.7	--	--	1.00 (0.90-1.00)	0 (0 – 0.95)	0.98 (0.86-1.00)
**Clinical diagnoses of foot disease**						
Ulcer type: neuropathic	83.7	0.55 (0.14)	Moderate	1.00 (0.86-1.00)	0.46 (0.20-0.74)	0.81 (0.64-0.91)
(Neuropathic or neuroisch.)						
Ulcer type is ischaemic	79.1	0.57 (0.13)	Moderate	0.74 (0.48-0.90)	0.83 (0.62-0.95)	0.78 (0.52-0.93)
(Neuroisch. or ischaemic)						
Combined surface area mm^2^#		0.99 (0.99–1.00)	Strong			
Any clinical infection	81.4	0.58 (0.13)	Moderate	0.71 (0.42-0.90)	0.86 (0.67-0.95)	0.71 (0.42-0.90)
UTWCS (Grade 1A)	81.4	0.61 (0.12)	Substantial	0.86 (0.56-0.97)	0.79 (0.60-0.91)	0.69 (0.41-0.86)
Deep ulcer	93.0	0.73 (0.15)	Substantial	0.71 (0.30-0.95)	0.97 (0.84-1.00)	0.83 (0.36-0.99)
(Grade 2 or 3)^						
**Clinical management principles performed**						
Ulcer debridement is required	95.3	0.39 (0.22)	Fair	1.00 (0.89-1.00)	0.50 (0.03-0.97)	0.98 (0.86-1.00)
Dressing is optimum	97.7	--	--	0.98 (0.86-1.00)	--	1.00 (0.90-1.00)
Antibiotics are required	90.7	0.76 (0.11)	Substantial	0.75 (0.43-0.93)	0.97 (0.81-1.00)	0.90 (0.54-0.99)
Off-loading is optimum	79.1	0.50 (0.14)	Moderate	0.80 (0.44-0.96)	0.79 (0.60-0.90)	0.53 (0.27-0.78)
Footwear is optimum	79.1	0.47 (0.15)	Moderate	0.70 (0.35-0.92)	0.82 (0.64-0.92)	0.54 (0.26-0.80)
Educated patient	100	--	--	1.00 (0.90-1.00)	--	1.00 (0.90-1.00)

K = Kappa; (SE) = Standard Error; % = Percentage agreement; PPV = Positive Predictive Value.

-- = Measure of agreement was unable to be calculated because at least one variable was a constant.

GP = General Practitioner; CVD = Cardiovascular Disease; ESRF = End Stage Renal Failure; PAD = Peripheral Arterial Disease; UTWCS = University of Texas Wound Classification System.

*= Weighted Kappa used for ordinal data.

# = Intraclass correlation (ICC) (Standard Deviation (SD)) for continuous data.

^ = Depth was determined from the UTWCS score.

#### Inter-rater reliability

Tables 4, 5 and 6 display the inter-rater reliability results for all three tests. Ninety-six different tests were able to be statistically tested for inter-rater reliability across the three different pairs of clinician agreement. Twenty four items (25%) recorded near perfect categories of agreement, 43 (45%) substantial/strong categories, 17 (18%) moderate categories and 12 (12%) weak/fair categories. Thus, overall 84 (88%) of these items recorded at least moderate categories of reliability (K > 0.4; ICC > 0.75). The items recording weak categories of reliability across two or more agreements tested included other (non-listed) condition, previous foot ulcer and optimum footwear.

**Table 6:** **Reliability measure of agreement summary statistics**: **Inter**-**rater** (**Senior clinician & clinician**) & **intra**-**rater** (**Clinician**)

	**Inter-rater reliability**			**Intra-rater reliability**		
**QHRFF field**	**%**	***K *** **(SE)**	**Strength of agreement [**[37]**]**	**%**	***K *** **(SE)**	**Strength of agreement [**[37]**]**
**General demographics**						
Indigenous status*	83.7	--	--	100	1.00 (0.01)	Near Perfect
**Health professionals attending (in the past week)**						
Podiatrist	88.4	--	--	N/A	N/A	N/A
GP	81.4	0.45 (0.15)	Moderate	N/A	N/A	N/A
Nurse	81.4	0.47 (0.14)	Moderate	N/A	N/A	N/A
Orthotist	100	--	--	N/A	N/A	N/A
Physician	90.7	--	--	N/A	N/A	N/A
Surgeon	90.7	0.45 (0.23)	Moderate	N/A	N/A	N/A
Other	97.7	--	--	N/A	N/A	N/A
**Medical co**-**morbidity history**						
Medical (diabetes) history	95.3	0.94 (0.05)	Near perfect	100	1.00 (0.01)	Near Perfect
Diabetes duration#		0.94 (0.87–0.97)	Strong		1.00 (0.99–1.00)	Strong
Recent BGLs >15 mmol/L*	83.7	0.67 (0.12)	Substantial	N/A	N/A	N/A
HbA1c result#		1.00 (1.00–1.00)	Strong		1.00 (1.00–1.00)	
Hypertension	88.4	0.73 (0.11)	Substantial	89.5	0.78 (0.15)	Substantial
Dyslipidaemia	83.7	0.67 (0.11)	Substantial	94.7	0.90 (0.10)	Near Perfect
Smoker	88.4	0.64 (0.15)	Substantial	94.7	0.83 (0.17)	Near Perfect
CVD	88.4	0.76 (0.10)	Substantial	84.2	0.69 (0.17)	Substantial
CKD	86.1	0.58 (0.15)	Moderate	94.7	0.86 (0.14)	Near Perfect
ESRF	97.7	0.66 (0.32)	Substantial	100	--	--
Other (non-listed)	69.1	0.34 (0.11)	Fair	78.9	0.57 (0.19)	Moderate
**High-risk history**						
Neuropathy	90.7	0.66 (0.16)	Substantial	94.7	--	--
PAD	83.7	0.67 (0.11)	Substantial	78.9	0.60 (0.16)	Moderate
Previous foot ulcer	90.7	0.72 (0.13)	Substantial	94.7	0.64 (0.33)	Substantial
Current foot ulcer	97.7	--	--	100	--	--
New foot ulcer	97.4	--	--	100	--	--
Previous amputation	97.7	0.95 (0.05)	Near perfect	100	1.00 (0.01)	Near Perfect
**Clinical diagnoses of high-risk factors**						
Neuropathy	97.7	0.90 (0.10)	Near perfect	94.7	--	--
PAD*	97.9	0.97 (0.03)	Near perfect	84.2	0.69 (0.16)	Substantial
Acute charcot	100	1.00 (0.01)	Near perfect	100	--	--
Foot deformity	83.7	0.38 (0.18)	Fair	84.2	0.31 (0.30)	Fair
Risk classification	100	--	--	100	--	--
**Clinical diagnoses of foot disease**						
Ulcer type*	88.4	0.81 (0.08)	Near perfect	84.2	0.77 (0.14)	Substantial
Combined surface area mm^2^#		0.94 (0.89–0.97)	Strong		1.00 (1.00–1.00)	Strong
Clinical signs infection*	76.7	0.60 (0.12)	Moderate	89.5	0.46 (0.32)	Moderate
UTWCS grade#		0.90 (0.83–0.95)	Strong		0.98 (0.94–0.99)	Strong
Ulcer depth^	95.3	0.90 (0.10)	Near perfect	94.7	0.79 (0.23)	Substantial
**Clinical management principles performed**						
Debrided ulcer/callus*	97.7	0.66 (0.32)	Substantial	94.7	--	--
Dressing optimum*	97.7	--	--	100	--	--
Antibiotics required*	79.1	0.54 (0.12)	Moderate	89.5	0.44 (0.33)	Moderate
Off-loading optimum*	72.1	0.36 (0.13)	Fair	68.4	0.32 (0.23)	Fair
Footwear optimum*	67.4	0.33 (0.14)	Fair	68.4	0.37 (0.21)	Fair
Educated patient*	97.7	--	--	89.5	--	--

K = Kappa; (SE) = Standard Error; -- = Measure of agreement was unable to be calculated because at least one variable was a constant.

GP = General Practitioner; CVD = Cardiovascular Disease; ESRF = End Stage Renal Failure; N/A = Not applicable to test due to unstable rating environment; PAD = Peripheral Arterial Disease; UTWCS = University of Texas Wound Classification System.

*= Weighted Kappa used for ordinal data;

# = Intraclass correlation (ICC) (Standard Deviation (SD)) for continuous data.

^ = Depth was determined from the UTWCS score.

#### Intra-rater reliability

Table 6 also displays the intra-rater reliability results for the level 3 clinician. The median (interquartile range) period between the first and second ratings for the intra-rater reliability testing was 2(1–2) weeks. Twenty-three items were able to be statistically tested. Six items (26%) recorded near perfect categories of agreement, ten (43%) substantial/strong categories, four (17%) moderate categories and three (13%) weak/fair categories. Thus, overall 20 (87%) of these items recorded at least a moderate category of reliability (K > 0.4; ICC > 0.75). Those items scoring weak categories of agreement were foot deformity, optimal offloading and optimal footwear.

## Discussion

The QHHRF appears to be the first multi-item tool developed and tested to identify multiple high-risk factors and foot disease complications in multiple at risk populations. Our findings indicate that the majority of the tool’s items demonstrates at least moderate categories of validity (face, content and criterion validity) and reliability (inter-rater and intra-rater); particularly those in the domains of identifying relevant medical co-morbidity history, and clinical diagnoses of high-risk factors and foot disease complications. However, some items in the domains of identifying different health professionals previously attending the patient and general clinical management principles performed appear to have weaker categories of validity and reliability that need addressing in future versions of the tool.

The QHRFF went through a number of developmental, validity and reliability tests to determine its status as a valid and reliable tool. The magnitude of validity (or accuracy) in this study was evaluated via the methods of face, content and importantly criterion validity. Face and content validity are considered the least robust of the validity tests due to their inherent subjectivity [31,33]. However, they are considered important factors in the development phase to ensure the tool can actually measure the general identified construct [31,33]; in this case foot disease. At the completion of phase one, the expert panel and network stakeholders endorsements implied high practicality, face and content validity.

Criterion validity is considered to be the most objective validity test [31,33]. To test concurrent criterion validity, as used by this study, a satisfactory criterion measure must be used. In this study the criterion measure to test criterion validity was an expert’s clinical diagnosis. Overall, the 'expert’ criterion measure used for this study were rated as having at least moderate categories of reliability for all but three items; any other (non-listed) co-morbidity, optimum footwear and UTWCS grade. The other (non-listed) co-morbidity and optimum footwear items rated in the weak categories in most other inter- or intra-rater reliability agreement tested and should be reviewed or removed in future versions of the QHRFF. The weak-moderate agreement for the UTWCS grade was unexpected as this tool has been validated extensively in the past [62], however, all other inter- or intra-rater agreements on the UTWCS were rated as substantial/strong categories of reliability. Thus, it is recommended that the UTWCS grade be retained in future with more emphasis applied on the UTWCS grading system in the training sessions provided. It is certainly possible that the criterion measure for each item may have been more robust if the individual gold standard test for each individual item were used; for example an angiogram to diagnose peripheral arterial disease [22] or nerve conduction studies to diagnose neuropathy [44]. However, such an approach would have been particularly resource and time intensive and was thus not practical within the resources available to this study.

The QHRFF items were tested for concurrent criterion validity by using two different blinded representative general clinicians’ ratings compared to the criterion measure (a reliable expert’s diagnosis) on the same patients. Positive predictive values from this testing indicated the majority of QHRFF items had at least moderate validity when used by clinicians with different levels of expertise. The high positive predictive values suggest that the proportion of people with a positive test result for individual QHRFF items actually had the relevant medical co-morbidity, high-risk factor or foot disease complication of interest. Although there were a few notable exceptions such as identifying different health professionals attending previously, recent blood glucose levels (BGLs) > 15 mmol/L, ESRF, optimal offloading and optimum footwear. However, overall the QHRFF tool appears to demonstrate acceptable validity in the majority of its items to be considered a valid tool to test the foot disease construct in multiple at risk populations.

Reliability (or consistency) is a major prerequisite of any useful items to be measured [31,33]. In this study inter-rater and intra-rater measures of agreement were used for reliability. Inter-rater reliability was primarily assessed using three clinicians with different representative levels of expertise in managing foot disease. Overall, all items consistently displayed at least moderate categories of reliability, except for any other (non-listed) co-morbidity, optimum footwear and previous foot ulcer. Optimum footwear was also identified to have weak categories of intra-rater reliability; along with identifying foot deformity and optimum offloading. Thus, the QHRFF tool appears to demonstrate satisfactory reliability to collect the majority of items in the foot disease construct. However, the authors recognise that it would have been preferable to test reliability with more clinicians across a larger sample of patients, yet this was beyond the resourcing available for this study.

Notwithstanding, the aforementioned methodological limitations, the study does incorporate many best practice research design methods for testing validity and reliability. These methods included testing the reliability of the criterion measure to diagnose and measure each item, testing the subsequent concurrent criterion validity of representative clinicians, and testing the reliability of the tool when used by clinicians with different levels of expertise. Furthermore, the study employed patient samples that were highly representative of the demographics and co-morbidity prevalence rates found in multiple 'at risk’ populations for high risk factors and foot disease complications. These high prevalence rates not only suggest the tool was tested in the construct it was designed to measure, but literature suggests higher prevalence rates improve the statistical robustness of validity and reliability results [31]. Lastly, many existing validated single-item tools [23,46,60,62] were incorporated within the QHRFF tool and this potentially adds weight to the validity reported in this study. Thus, overall the QHRFF tool appears to be a valid and reliable tool to collect the vast majority of items contained within the foot disease construct and can be used as a general tool to complement existing specific tools in the field of foot disease.

### Limitations

Several limitations have already been outlined in this study including the use of a general criterion measure of an experts’ clinical diagnoses for each item, only using a limited number of representative clinicians and testing intra-rater reliability on one clinician in a small sample. Other limitations include: not performing a systematic literature review (and thus some evidence based items and tools may have been overlooked); using only podiatrists as the clinical raters; using historically defined strength categories for validity and reliability; not testing the tool for construct validity, predictive validity, or factor analysis; and the settings used were existing HRFS only. It is recommended that any future research into this tool should address these methodological limitations by investigating the factor analysis, construct and predictive criterion validity of the tool’s items in a much larger and broader sample of patients with more multi-disciplinary clinician raters. Furthermore, if resources permit, the use of individual gold standard criterion measures and/or existing concurrent validated single-item tools should be considered.

However, there are a number of recommendations from this study’s findings that should be considered for implementation in future versions of the QHRFF tool. Firstly, those items reporting weaker categories of validity or reliability and that potentially collect duplicate information to other more reliable items should be removed; including any other (non-listed) co-morbidity, combined surface area (change), recent BGLs greater than 15 mmol/L and new ulcer. Secondly, some items’ definitions should be reviewed in an attempt to improve the item’s future reliability and validity. For example, previous foot ulcer definition could be modified to identify a previously “healed” foot ulcer, 'health professionals attending’ previously requires an exact retrospective time period that patients were in attendance in alignment with similar literature (such as “in the previous two weeks”) [64,65] and optimum offloading should have a more explicit definition referring directly to non-removable offloading definitions in the existing literature [14]. Thirdly, the validation of criteria for identifying a foot deformity and optimum footwear are urgently required for these 'at risk’ populations. To the best of the author’s knowledge such tools for these items have been developed [14,50] yet are to be validated and this study was no exception. However, in the meantime the authors recommend adding the numerical foot deformity score to the QHRFF to improve accuracy and test for validity. Lastly, a systematic literature review for other reported independent associates for foot disease (such as cancer, arthritis, depression, trauma, vision impairment, mobility impairment and social determinant factors) and other outcome measures for foot disease (such as quality of life measures) should be performed to ensure all 'at risk’ populations and outcome measures for foot disease are identified, considered and potentially included in the next QHRFF version.

## Conclusions

The QHRFF tool appears to be the first multi-item tool developed and tested to identify multiple high-risk factors and foot disease complications in multiple at risk populations. Acceptable validity and reliability were demonstrated for the majority of items; particularly in the domains of identifying relevant medical co-morbidity history and clinically diagnosing high-risk factors and foot disease complications. However, recommendations to remove, add or redefine items registering weak validity or reliability scores should be implemented to improve future versions of the QHRFF tool; particularly in the domains of health professionals attending and clinical management principles. Overall, the QHRFF tool appears to demonstrate the practicality, validity and reliability required to facilitate robust clinical assessment and data capture to measure the large burden of foot disease facing our multiple at risk populations.

## Abbreviations

ANOVA: Analysis of variance; BGL: Blood glucose levels; CINAHL: Cumulative index to nursing and allied health literature; CKD: Chronic kidney disease; DFF: Diabetic foot form; ESRF: End stage renal failure; GP: General practitioner; HRFS: High risk foot service; ICC: Intra-class correlation; K: Kappa; wK: Weighted Kappa; MEDLINE: Medical literature analysis and retrieval system online; PPV: Positive predictive value; QHRFF: Queensland high risk foot form; SD: Standard deviation; SE: Standard error; UTWCS: University of Texas wound classification system.

## Competing interests

The authors have no relevant conflict of interest to disclose.

## Authors’ contributions

PAL conceived, designed, researched data, contributed to discussion, wrote and reviewed/edited the manuscript. VN provided research assistance, researched data, contributed to discussion and reviewed/edited the manuscript. EMK conceived, contributed to discussion and reviewed/edited the manuscript. MSK, SSK and LR designed, contributed to discussion, and reviewed/edited the manuscript. CH designed, researched data and reviewed/edited the manuscript. All authors read and approved the final manuscript.
